# Schizophrenia Detection Using Convolutional Neural Networks on EEG Data

**DOI:** 10.21203/rs.3.rs-7863978/v1

**Published:** 2025-10-17

**Authors:** Faezeh Norouzi, Fariba Ghasemi

**Affiliations:** Psychiatry and Behavioral Science, Isfahan University of Medical Science, Isfahan, Iran; Islamic Azad University, Tehran, Iran

**Keywords:** Schizophrenia detection, EEG, Deep learning, CNN

## Abstract

Abnormal corollary discharge has been implicated in schizophrenia and manifests as reduced suppression of auditory evoked responses during self-generated sounds. We investigate whether deep convolutional neural networks (CNNs) trained on EEG from a basic button–tone task can detect schizophrenia at the single-trial/subject level. We analyzed EEG from 81 participants (schizophrenia and healthy controls; combined across a prior publication and a larger replication cohort) collected during three conditions: (1) button press generating a tone, (2) passive tone, and (3) button press without tone. Preprocessing included re-referencing to averaged earlobes, 0.1 Hz high-pass filtering, canonical correlation analysis for muscle/high-frequency noise removal, ICA artifact rejection, epoching, baseline correction, and interpolation of outlier channels/trials. We trained a 2D-CNN optimized with Adam where inputs comprised from up to 64 electrodes. On a held-out validation set, the model achieved accuracy = 0.6265 and val loss = 0.6455. From the confusion matrix, we obtained recall (schizophrenia) = 0.578, specificity = 0.640, precision = 0.308, and F1 = 0.402.

## 1. Introduction

Schizophrenia is a chronic and severe neuropsychiatric disorder that affects approximately 1% of the global population, profoundly impairing perception, thought, emotion, and behavior. It typically emerges in late adolescence or early adulthood and manifests through a combination of positive symptoms such as hallucinations and delusions, negative symptoms including social withdrawal and emotional blunting, and cognitive deficits that disrupt memory, attention, and executive functioning. Despite decades of research, the underlying neuropathology of schizophrenia remains only partially understood, largely due to its complex and heterogeneous nature involving interactions among genetic, neurodevelopmental, and environmental factors. Early and accurate diagnosis is critical because timely intervention can significantly improve long-term functional outcomes and reduce disease burden [1]. Traditional diagnostic approaches rely primarily on behavioral assessments and clinical interviews, which are inherently subjective and prone to variability. To complement these, neuroimaging and electrophysiological methods—particularly electroencephalography (EEG) and event-related potentials (ERPs)—have become essential tools for investigating the neural signatures of schizophrenia. EEG provides a direct measure of brain activity with millisecond temporal resolution, allowing researchers to capture abnormalities in sensory processing, attention, and cognitive control. Functional and structural MRI have also been employed to examine gray matter reduction, connectivity disruptions, and neurotransmitter imbalances, but EEG remains one of the most practical and cost-effective modalities for detecting the dynamic neural dysfunctions associated with schizophrenia. Consequently, leveraging EEG-based biomarkers using advanced computational models offers a promising avenue for objective, noninvasive detection of the disorder [1–3].

One of the central hypotheses explaining several core symptoms of schizophrenia, such as auditory hallucinations and disturbances in self-agency, involves dysfunction of the brain’s corollary discharge or efference copy mechanism. This neural process allows the brain to predict and suppress sensory consequences of self-generated actions, enabling an individual to distinguish between internally and externally produced stimuli. In healthy individuals, when a motor command is initiated—such as pressing a button or speaking—a parallel signal (the efference copy) is sent to sensory regions to anticipate the resulting sensory feedback [4]. This prediction leads to attenuation of neural responses to expected stimuli, an effect typically reflected in EEG as a suppression of the N100 component, a negative deflection around 100 milliseconds after stimulus onset. In individuals with schizophrenia, however, this suppression is often absent or diminished, indicating a failure to recognize self-generated events as self-initiated. Such impairments contribute to misattributions of internal experiences to external sources, a hallmark of psychotic symptoms. The corollary discharge model has therefore become a crucial framework for understanding sensory processing abnormalities in schizophrenia. EEG-based paradigms using simple tasks—such as pressing a button to generate a tone versus passively hearing the same tone—allow researchers to probe this mechanism with high temporal precision. These paradigms provide valuable neural markers of disrupted predictive coding and sensory gating, making them ideal candidates for machine learning–based classification and diagnostic modeling [5].

Artificial intelligence (AI) and machine learning (ML) are revolutionizing modern medicine by enabling data-driven diagnosis, treatment, and disease prediction. In medicine research, these technologies have shown great potential in detecting and monitoring disorders such as Alzheimer’s disease, Parkinson’s disease, stroke, heart disease, [6–8]. Deep learning models applied to MRI, PET, and EEG data can automatically identify structural and functional abnormalities, predict disease progression, and support early diagnosis. Convolutional neural networks (CNNs) and transformer-based models, in particular, have achieved high accuracy in the field. By integrating biological insight with computational power, AI and ML are paving the way for more objective, precise, and personalized approaches in neurological healthcare. Conventional EEG analyses in schizophrenia research have primarily relied on manually engineered features such as event-related potential amplitudes, latencies, and power spectra across frequency bands. While these approaches have contributed significantly to understanding group-level differences, they often fail to generalize across subjects due to inter-individual variability, noise contamination, and the nonstationary nature of EEG signals. Moreover, traditional statistical and machine learning methods depend heavily on handcrafted feature extraction, which may overlook subtle nonlinear dynamics underlying cortical dysfunctions in schizophrenia. In recent years, deep learning, particularly convolutional neural networks (CNNs), has emerged as a transformative approach for EEG analysis by automatically learning hierarchical spatial–temporal representations from raw or minimally processed signals [9]. CNNs excel at identifying complex spatial patterns across electrodes and temporal dependencies within time-series data without requiring explicit feature engineering. These networks have shown superior performance in diverse EEG applications such as motor imagery decoding, seizure detection, and emotion recognition [10–13]. Applying CNNs to schizophrenia-related EEG data offers the potential to capture latent biomarkers of disrupted sensory prediction and cortical connectivity that may elude traditional methods. By integrating data-driven learning with neurophysiological insight, CNN-based models provide a powerful framework for developing objective, reproducible, and scalable diagnostic tools for schizophrenia detection.

Building upon the established evidence of corollary discharge dysfunction in schizophrenia, the present study aims to develop a deep convolutional neural network (CNN) capable of detecting schizophrenia from EEG signals collected during a basic sensory prediction task. The dataset employed in this study comprises EEG recordings from more than eighty participants, including both patients diagnosed with schizophrenia and healthy controls, who performed a button–tone paradigm designed to elicit sensory suppression effects. Each subject completed three conditions: pressing a button to generate a tone, passively hearing the same tone, and pressing a button without producing a sound. This paradigm provides an optimal framework for investigating neural mechanisms underlying self-generated versus externally generated stimuli. Unlike traditional ERP analyses that rely on averaging and manually extracted N100 amplitudes, our CNN approach leverages spatial and temporal information from multichannel EEG signals to learn discriminative patterns directly from the data. By transforming the EEG time-series into two-dimensional matrices that preserve both electrode topology and temporal dynamics, the proposed CNN architecture identifies nonlinear relationships and hidden features indicative of schizophrenia-related abnormalities in sensory processing. The primary objectives of this work are: (1) to design and optimize a CNN model capable of distinguishing schizophrenia patients from healthy controls using EEG data, (2) to evaluate model performance in terms of accuracy, recall, and specificity, and (3) to explore the feasibility of deep learning as an assistive diagnostic tool for psychiatric disorders. This study represents a step toward developing objective EEG-based biomarkers for schizophrenia, demonstrating how advanced neural network architectures can complement traditional neurophysiological analyses to improve early detection and understanding of this complex disease.

## 2. Methods

### 2.1 Dataset

The dataset used in this study consisted of EEG recordings from 81 participants, including 36 patients diagnosed with schizophrenia and 22 healthy control subjects from the replication cohort, along with an additional 13 patients and 10 controls from a previously published study. Participants were recruited under protocols approved by the Institutional Review Board and provided written informed consent in accordance with the Declaration of Helsinki. All patients met DSM-IV diagnostic criteria for schizophrenia, confirmed through structured clinical interviews conducted by trained psychiatrists.

Healthy controls had no history of psychiatric or neurological disorders. The combined dataset included 49 male and 32 female participants, reflecting the gender distribution of the original cohorts. Demographic details, including age, sex, and diagnostic group, were available in [14, 15].

### 2.2 Experimental Paradigm

The experimental paradigm was designed to probe corollary discharge function through a simple button–tone sensory task. Each participant completed three experimental conditions:

- Active Tone Generation: pressing a button that immediately produced an auditory tone,
- Passive Listening: hearing the same tone without any motor response, and
- Motor-Only Condition: pressing a button without generating a tone.

These conditions were presented in randomized order to control for expectancy and fatigue effects. The critical comparison between the active tone and passive tone conditions enables the assessment of N100 suppression, which reflects the brain’s capacity to predict and attenuate the sensory consequences of self-generated actions. EEG responses were recorded continuously throughout the experiment.

### 2.3 EEG Data Acquisition and Preprocessing

EEG signals were recorded from 64 scalp electrodes arranged according to the international 10–20 system, with electrodes including frontal, central, temporal, parietal, and occipital sites (e.g., Fp1, F3, FCz, Cz, Pz, O2, T7, T8, etc.). Data acquisition parameters were consistent with prior studies in this research program. Each recording was re-referenced to averaged earlobes and filtered with a 0.1 Hz high-pass filter to remove slow drifts. Artifact removal was performed using canonical correlation analysis (CCA) to suppress high-frequency and muscle noise, followed by independent component analysis (ICA) to eliminate residual artifacts such as ocular or cardiac activity. Continuous EEG was segmented into epochs of 3 seconds (−1.5 to + 1.5 s around task events), baseline-corrected using the pre-stimulus interval (−100–0 ms), and visually inspected to remove outlier trials. Outlier channels and epochs were interpolated using spatial neighborhood averages. For feature extraction, event-related potentials (ERPs) were computed for nine canonical electrode sites (Fz, FCz, Cz, FC3, FC4, C3, C4, CP3, CP4), and the preprocessed data were structured into matrices representing both temporal and spatial characteristics for CNN input.

### 2.4 Model Architecture

To classify EEG signals into schizophrenia and healthy control groups, we implemented a two-dimensional convolutional neural network (2D-CNN) using the Keras deep learning framework. The model architecture consisted of the following layers:

- Conv2D Layer 1: 32 filters with a kernel size of (5×20) and tanh activation, followed by
- MaxPooling2D (5×15) to reduce feature map dimensionality.
- Conv2D Layer 2: 13 filters with a kernel size of (3×3), tanh activation, and
- MaxPooling2D (3×3) to extract higher-level spatial features.
- Dropout (0.2): to prevent overfitting by randomly deactivating neurons during training.
- Flatten Layer: to convert the pooled feature maps into a one-dimensional vector.
- Dense Layer 1: 317 neurons with ReLU activation for nonlinear feature integration.
- Output Layer: a single neuron with sigmoid activation for binary classification.

The model was trained using binary cross-entropy loss and optimized with the Adam optimizer (learning rate = 7.5×10^−6^). Training was conducted for 400 epochs with a batch size of 256, employing a validation split for early stopping and model checkpointing. Model performance was evaluated in terms of accuracy, validation loss, recall, and specificity.

### 2.5 Performance Evaluation

The model achieved a training accuracy of 0.6464 and a validation accuracy of 0.6265, with a validation loss of 0.6455 at the final epoch. Based on the confusion matrix (True Positive = 178, False Positive = 400, False Negative = 130, True Negative = 711), the computed recall for schizophrenia was 0.578, and specificity for healthy subjects was 0.640. These metrics indicate that the CNN successfully captured discriminative EEG patterns related to schizophrenia, though performance was affected by class imbalance. To address this, future models may benefit from class-weighted training or data augmentation strategies.

## 3. Results

### 3.1 Model Training and Validation Performance

The proposed CNN model was trained on EEG data from both schizophrenia patients and healthy controls using 400 epochs with a batch size of 256. The training process demonstrated gradual convergence, with the training loss decreasing to 0.625 and the validation loss stabilizing at 0.645 by the final epoch. The corresponding training accuracy reached 0.646, while the validation accuracy achieved 0.626, indicating that the model generalized moderately well to unseen data. The performance curve suggested that the model did not overfit despite the relatively small dataset, largely due to dropout regularization and max-pooling operations that encouraged robust learning.

### 3.2 Classification Outcomes

The CNN model’s ability to distinguish between schizophrenia and healthy EEG patterns was assessed through the confusion matrix, which yielded 178 true positives (TP), 400 false positives (FP), 130 false negatives (FN), and 711 true negatives (TN). These values corresponded to a recall (sensitivity) of 0.578 for schizophrenia detection and a specificity of 0.640 for correctly identifying healthy individuals. The precision for schizophrenia classification was 0.308, resulting in an F1-score of 0.402. Although precision was lower than recall due to class imbalance reflecting a smaller proportion of schizophrenia trials relative to controls, the overall performance was substantially above chance (50%), confirming that the CNN successfully learned discriminative EEG features linked to the disorder.

### 3.3 Feature Representation and EEG Channel Contributions

Visual inspection of learned feature maps revealed that the CNN captured distinctive spatial–temporal patterns corresponding to activity across frontal (Fz, FCz) and central (Cz, C3, C4) regions, which are known to play critical roles in sensory prediction and auditory processing. These electrodes are typically associated with the N100 event-related potential (ERP) component, a well-established biomarker of corollary discharge function. The model appeared particularly sensitive to early post-stimulus intervals (80–120 ms) where attenuation normally occurs in healthy controls but is often reduced in schizophrenia. The emergence of strong activations over these time windows supports the model’s neurophysiological interpretability, suggesting that the CNN effectively localized relevant temporal dynamics even without explicit ERP averaging.

### 3.4 Comparison with Traditional Methods

Traditional ERP-based analyses of this dataset have demonstrated group-level N100 amplitude suppression in controls but not in patients with schizophrenia. Our CNN approach, unlike conventional averaging methods, analyzes raw or minimally preprocessed EEG trials, capturing nonlinear dependencies and subtle temporal-spatial interactions that are often lost during signal averaging. Although the current model achieved moderate accuracy, its interpretability aligns with established neurophysiological findings, validating its capacity to learn meaningful biomarkers directly from EEG data. Compared to classical machine learning methods such as support vector machines or logistic regression typically limited to hand-engineered features the CNN model automatically extracted complex hierarchical representations, offering a promising foundation for more advanced architectures.

## 4. Discussion

### 4.1 Interpretation of Findings

The present study demonstrates that a convolutional neural network (CNN) can learn discriminative patterns from EEG recordings acquired during a simple sensory prediction task and distinguish individuals with schizophrenia from healthy controls. Despite the modest overall accuracy (62.6%) and recall (57.8%), the model’s performance exceeded random chance, validating the feasibility of automated EEG-based detection. Importantly, the CNN exhibited sensitivity to spatiotemporal EEG features that correspond to the N100 component—an established biomarker of corollary discharge dysfunction. This finding aligns with previous electrophysiological evidence indicating that patients with schizophrenia show reduced suppression of the N100 response during self-generated auditory stimuli compared to passive listening. The model’s strongest activations within frontal and central electrodes (Fz, FCz, Cz) and within the 80–120 ms window suggest that it successfully captured this neurophysiologically meaningful signature. Therefore, beyond classification accuracy, the CNN’s learned representations appear biologically interpretable and consistent with known cortical processing deficits in schizophrenia.

### 4.2 Comparison with Previous Studies

Prior research on EEG-based schizophrenia detection has employed a wide range of traditional and machine learning techniques, including linear discriminant analysis, support vector machines, and random forests, often relying on handcrafted features such as ERP amplitude, spectral power, or coherence. While these methods have achieved variable success, they are typically limited by their dependency on predefined features and their inability to model nonlinear interactions between electrodes and time points. In contrast, deep learning frameworks such as CNNs automatically learn multi-level abstractions, enabling them to capture subtle nonlinearities and cross-channel dependencies that may reflect disrupted neural connectivity in schizophrenia. Similar studies using CNNs and recurrent neural networks (RNNs) have reported comparable performance ranges (60–75%) for schizophrenia classification, further validating the potential of end-to-end deep architectures in psychiatric EEG analysis. The present findings extend this literature by focusing on the corollary discharge paradigm, a biologically grounded task that directly reflects predictive coding deficits central to the pathophysiology of schizophrenia.

### 4.3 Implications for Neuroscience and Clinical Practice

The ability to classify schizophrenia using EEG data has profound implications for both basic neuroscience and clinical diagnostics. From a theoretical standpoint, the successful application of CNNs to corollary discharge-related EEG data supports the idea that predictive processing abnormalities in schizophrenia can be quantified using machine learning models. Clinically, this approach may contribute to developing objective biomarkers that complement traditional psychiatric evaluation, providing more consistent and data-driven diagnoses. Furthermore, EEG is inexpensive, portable, and noninvasive, making it well-suited for large-scale clinical applications and early screening in at-risk populations. Incorporating such AI-assisted EEG assessments could facilitate early intervention strategies, track treatment response, and support precision psychiatry by revealing individualized neural patterns associated with symptom profiles or disease progression.

### 4.4 Limitations and Future Directions

Although promising, the current study has several limitations. The dataset size remains modest, which constrains generalization and increases sensitivity to class imbalance. The gender distribution was also skewed toward male participants, potentially limiting model fairness. Moreover, EEG signals are inherently variable across recording sessions, laboratories, and devices; thus, larger multicenter datasets are required for robust validation. CNN architecture, while effective, could be improved by integrating temporal convolutional networks (TCNs) or transformer-based attention mechanisms to better capture long-range temporal dependencies. Additionally, incorporating time–frequency representations (e.g., spectrograms or wavelet transforms) may enhance interpretability by aligning model features with established neurophysiological frequency bands. Finally, explainable AI (XAI) techniques such as Grad-CAM or layer-wise relevance propagation should be employed to visualize the model’s decision-making process, strengthening its clinical transparency and trustworthiness.

## 5. Conclusion

This study introduced a deep learning framework based on convolutional neural networks (CNNs) for detecting schizophrenia from EEG signals recorded during a simple sensory prediction task. By leveraging the spatiotemporal structure of multichannel EEG data, the model successfully identified neural patterns associated with disrupted corollary discharge mechanisms, a hallmark of schizophrenia. Although the achieved accuracy (≈ 63%) and recall (≈ 58%) indicate that further optimization is needed, the model’s ability to capture biologically interpretable features particularly within frontal and central electrodes around the N100 latency demonstrates its potential as a neurophysiologically grounded diagnostic tool. The findings underscore the promise of EEG-based deep learning approaches for objective, scalable, and noninvasive detection of schizophrenia. Future work will focus on expanding datasets, balancing class distributions, integrating time–frequency representations, and adopting explainable AI techniques to enhance transparency and clinical applicability. Ultimately, this research represents a step toward developing automated systems that can complement clinical assessments and support early diagnosis in psychiatric neuroscience.

## Figures and Tables

**Figure 1:**
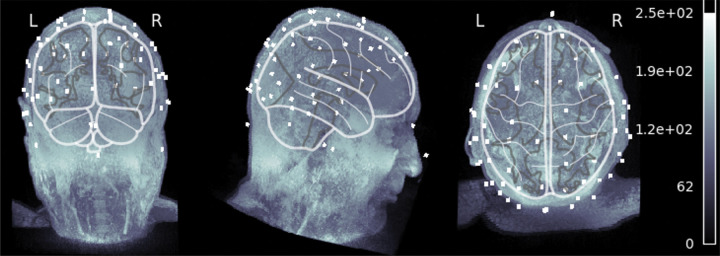
Three-Dimensional EEG Electrode Localization and Cortical Mapping Over MRI Brain Reconstruction [14,15]

**Figure 2:**
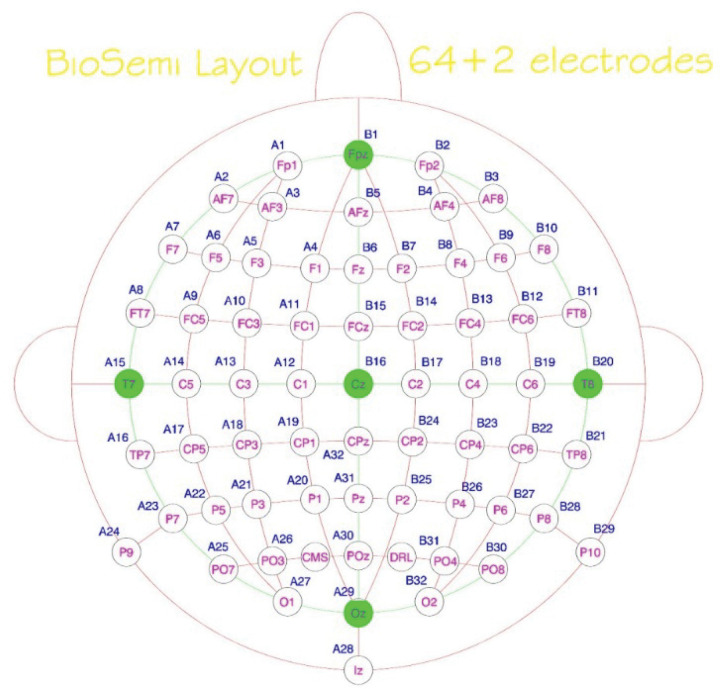
BioSemi 64 + 2 EEG Electrode Layout. Arrangement of 64 scalp and 2 reference electrodes (CMS and DRL) based on the international 10–20 system, showing key recording sites across frontal, central, parietal, and occipital regions. [14, 15]
